# Ultrasensitive Magnetic Field Sensing Based on Refractive-Index-Matched Coupling

**DOI:** 10.3390/s17071590

**Published:** 2017-07-07

**Authors:** Jie Rao, Shengli Pu, Tianjun Yao, Delong Su

**Affiliations:** 1 College of Science, University of Shanghai for Science and Technology, Shanghai 200093, China; 152251908@st.usst.edu.cn (J.R.); 162281924@st.usst.edu.cn (T.Y.); 142231886@st.usst.edu.cn (D.S.); 2 Shanghai Key Laboratory of Modern Optical System, University of Shanghai for Science and Technology, Shanghai 200093, China

**Keywords:** magnetic field sensor, magnetic fluid, singlemode-no-core-singlemode fiber structures, refractive-index-matched coupling

## Abstract

An ultrasensitive magnetic field sensor is proposed and investigated experimentally. The no-core fiber is fusion-spliced between two pieces of single-mode fibers and then immersed in magnetic fluid with an appropriate value of refractive index. Under the refractive-index-matched coupling condition, the guided mode becomes leaky and a coupling wavelength dip in the transmission spectrum of the structure is observed. The coupling wavelength dip is extremely sensitive to the ambient environment. The excellent sensitivity to the refractive index is measured to be 116.681 μm/RIU (refractive index unit) in the refractive index range of 1.45691–1.45926. For the as-fabricated sensors, the highest magnetic field sensing sensitivities of 6.33 and 1.83 nm/mT are achieved at low and high fields, respectively. The sensitivity is considerably enhanced compared with those of previously designed, similar structures.

## 1. Introduction

Magnetic fluid (MF) is a novel kind of functional material, which presents solid-magnetic-material-like magnetism and liquid-like fluidity. It consists of magnetic nanoparticles dispersed in a suitable liquid carrier. The magnetic nanoparticles are coated with surfactant to form a stable colloidal system [1]. The microstructures within MF are diverse under the external applied magnetic field. This will lead to many unique optical properties of MF under an applied magnetic field, such as tunable refractive index (RI), tunable birefringence, and tunable transmittance [2]. Until now, many optical devices and applications based on MF have been proposed and demonstrated, for example, optical gratings [3,4], optical switches [5], magnetic field sensors [1,6,7,8,9,10,11,12,13,14,15,16], tunable filters [17], tunable slow light [18], and couplers [19].

Recently, the singlemode-multimode-singlemode (SMS) or singlemode-no-core-singlemode (SNS) fiber structures have been designed to sense temperature, RI, chemical concentration, strain and magnetic field [1,6,20,21,22,23]. They are low-cost and easy to fabricate. The fundamental sensing principle is the multimode interference within the multimode fiber or no-core fiber (NCF). The sensitivity of these structures depends on the difference of response to ambient variation between the involved modes. On the other hand, refractive-index-matched coupling (RIMC) is attractive for widely tunable filtering and ultrasensitive sensing applications [24,25,26,27]. The fundamental principle is based on the material dispersion effect.

In this work, an MF with an appropriate RI value is utilized to cover the SNS fiber structure to realize magnetic field sensing with ultrahigh sensitivity. Different from the conventional SMS/SNS fiber structures, the sensing principle of this work is based on RIMC. At RIMC, the guided modes within the NCF become leaky, which yield a coupling wavelength dip (CWD) in the transmission spectrum. The CWD will shift significantly, and even the ambient environment changes slightly. Therefore, the sensitivity of the proposed magnetic field sensor can be markedly enhanced.

## 2. Sensor Structure and Sensing Principle

**Figure 1:**
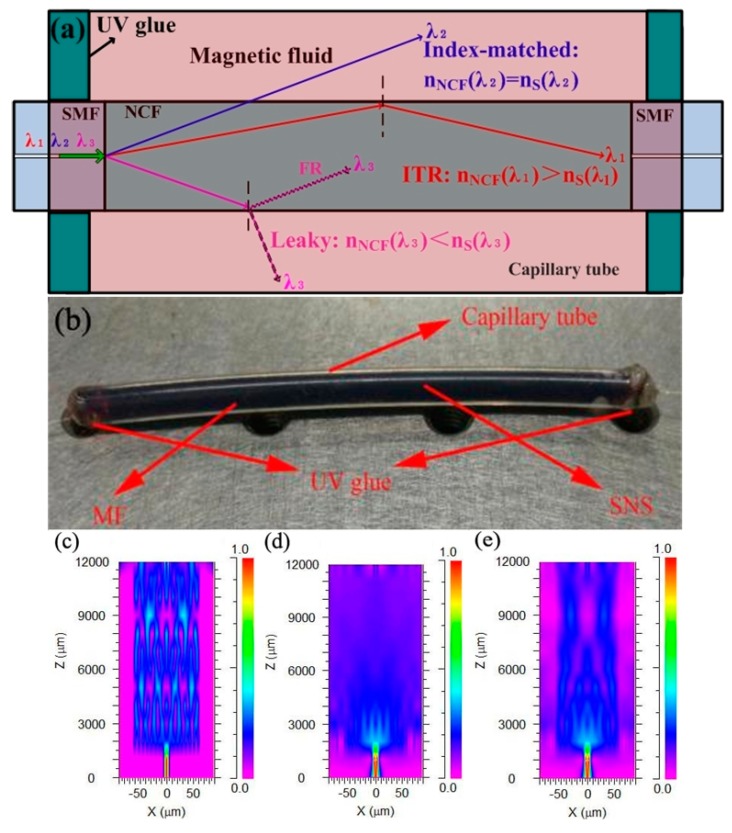
(**a**) Schematic of the proposed sensor; (**b**) picture of the as-fabricated sensor; (**c**) simulation of energy distribution within the no-core fiber (NCF) for the guided ($n_{NCF}(\lambda_{1})>n_{S}(\lambda_{1})$); (**d**) index-matched ($n_{NCF}(\lambda_{2})=n_{S}(\lambda_{2})$) and (**e**) leaky ($n_{NCF}(\lambda_{3})<n_{S}(\lambda_{3})$) cases, respectively.

Figure 1a shows the schematic diagram of the proposed sensor. A section of NCF with a diameter of 125 μm is fusion spliced between two pieces of singlemode fibers. The whole structure is placed in a capillary tube, which is filled with MF. Both ends of the capillary tube are sealed with UV glue to prevent the MF from leaking and evaporating. Therefore, the cladding of the NCF is actually the ambient MF. The as-fabricated sensor is shown in Figure 1b.

When the incident light is broadband and propagates within the NCF, there are three possible cases depending on the relative relationship between the RI of NCF and that of MF: guided, index-matched, and leaky (see Figure 1a). Due to the different dispersion properties of NCF and MF, their dispersion profiles may intersect at a certain wavelength range. The guided condition means that the internal total reflection (ITR) happens, i.e., $n_{NCF}(\lambda_{1})>n_{S}(\lambda_{1})$, where $n_{NCF}(\lambda_{1})$ and $n_{S}(\lambda_{1})$ are the RIs of NCF and MF at $\lambda_{1}$, respectively. At a specific wavelength (for example $\lambda_{2}$), $n_{NCF}(\lambda_{2})=n_{S}(\lambda_{2})$ and the index-matched condition is satisfied. This is the RIMC, which can cause a high loss at this specific wavelength. The leaky case implies $n_{NCF}(\lambda_{3})<n_{S}(\lambda_{3})$ and the Fresnel reflection (FR) will happen at this wavelength. Figure 1c–e) displays the simulations of energy distribution within the NCF for the three cases, respectively. It is clear from Figure 1c–e that the light is effectively confined within the NCF when $n_{NCF}(\lambda_{1})>n_{S}(\lambda_{1})$. Strong interference is obvious. Under the index-matched condition ($n_{NCF}(\lambda_{2})=n_{S}(\lambda_{2})$), the energy leaks out of NCF along with the light propagation. Meanwhile, for the FR case ($n_{NCF}(\lambda_{3})<n_{S}(\lambda_{3})$), part of energy returns to the fiber. Then, weak light is observed within the NCF as shown in Figure 1e.

The CWD is related with the index-match between the NCF and the surrounding media (MF) at a specific wavelength. The RI of the MF changes with the external magnetic field, but that of the NCF is independent of magnetic field. So, the index-matched wavelength will shift with the magnetic field. Due to the low dispersion of the NCF and MF, i.e., small slopes of the dispersion profiles, the index-matched wavelength will shift significantly, and even the ambient environment varies slightly. Thus, the magnetic field sensing with ultrahigh sensitivity can be realized through observing the shift of the CWD.

## 3. Experimental Results and Discussion

**Figure 2:**
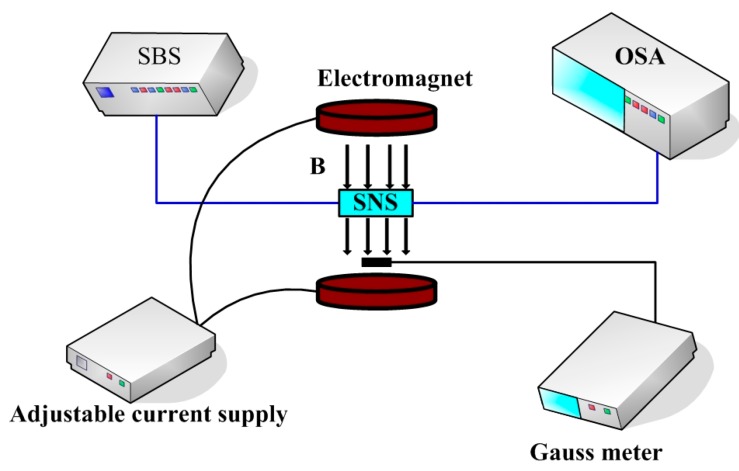
Schematic diagram of the experimental setup for investigating the sensing properties.

The schematic of the experimental setup for investing the sensing properties is shown in Figure 2. Light from the supercontinuum broadband source (SBS, Wuhan Yangtze Soton Laser Co., Ltd., Wuhan, China) is launched into the sensing structure and the output light is monitored and analyzed by an optical spectrum analyzer (OSA, Yokogawa AQ6370C, Tokyo, Japan). The magnetic field strength is adjusted by changing the magnitude of the supply current and calibrated by a gauss meter. The magnetic field direction is perpendicular to the optical fiber axis. During our experiments, the ambient temperature is kept constant.

**Figure 3:**
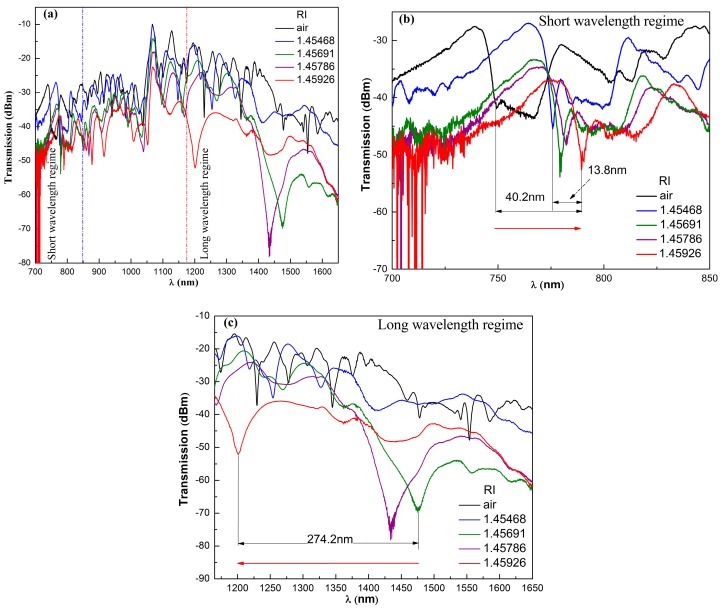
(**a**) Transmission spectra of the as-fabricated sensing structures, (**b**) the corresponding spectra at the short wavelength regime and (**c**) at the long wavelength regime.

The NCF with a diameter of 125 μm was provided by Yangtze Optical Fibre and Cable Joint Stock Limited Company (Wuhan, China). The RI of the oil-based MF (EXP08103, Ferrotec, Chiba, Japan) with volume fraction of 5.62% used in this work is measured to be 1.56403 by a refractometer (A670, Hanon, Jinan, China). For the easy occurrence of the abovementioned three cases, the initial MF is diluted with ethyl oleate to around 0.4% volume fraction, whose RI approaches that of the NCF. The diluted MFs with RIs of 1.45468, 1.45691, 1.45786, and 1.45926 (measured by the same refractometer) are obtained through finely tuning the volume fraction further, which are then utilized to fabricate the proposed sensing structures. The corresponding transmission spectra are shown in Figure 3a. Figure 3a indicates that the spectra for the MF-covered SNS structures is much smoother than that without the MF. This may be due to the RI of the MF approaching that of the NCF, which leads to the weakly guided case. More high-order modes radiate into the MF and become lossy, which weakens the modal interference effect. For clarity, the spectra at short and long wavelength regimes are replotted in Figure 3b,c respectively. Figure 3b shows that the spectrum red-shifts with the RI of the MF (see the red arrow). This is qualitatively consistent with that in [6]. Therefore, the mode interference dominates the spectrum at the short wavelength regime. The total wavelength shift is 40.2 nm. For the RI ranging from 1.45468 to 1.45926, the obtained wavelength shift is 13.8 nm and the corresponding average RI sensing sensitivity is 3013 nm/RIU (refractive index unit).

**Figure 4:**
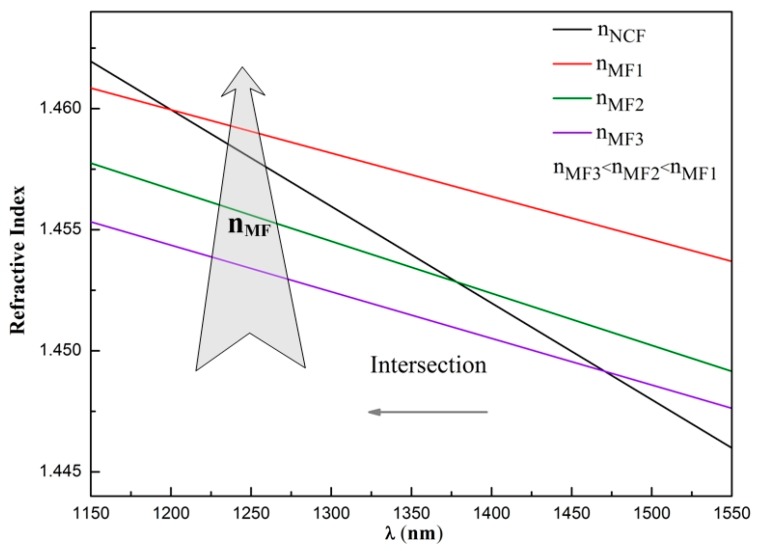
Dispersion profiles of the NCF and magnetic fluids (MFs) with different refractive indices (RIs).

At the long wavelength regime, there is a newly emerging dip wavelength occurring around 1480 nm when the RI of the MF is increased to 1.45691 (see Figure 3c). When the RI of the MF further increases, the newly emerging dip wavelength shifts toward the short wavelength side (see the red arrow in Figure 3c), which is opposite to the mode interference effect at the short wavelength regime. This newly emerging dip wavelength corresponds to the CWD. At this specific wavelength, the RI of the MF equals that of the NCF. To illustrate the shift of the CWD explicitly, Figure 4 schematically plots the dispersion profiles of the NCF and MFs with different RIs. The intersection point between the dispersion profiles of the NCF and MF corresponds to the CWD. Figure 4 clearly indicates that the intersection point shifts toward the short wavelength side as the RI of the MF increases. This is in agreement with the experimental results shown in Figure 3c.

At the long wavelength regime, the total wavelength shift is 274.2 nm in the range of 1.45691–1.45926, which corresponds to an average RI sensing sensitivity of 116.681 μm/RIU. Hence, the sensitivity based on the RIMC (leak of guided modes) is around 39 times higher than that based on mode interference. Thus, the proposed structure can be employed to realize a magnetic field sensor with ultrahigh sensitivity through interrogating the CWD.

**Figure 5:**
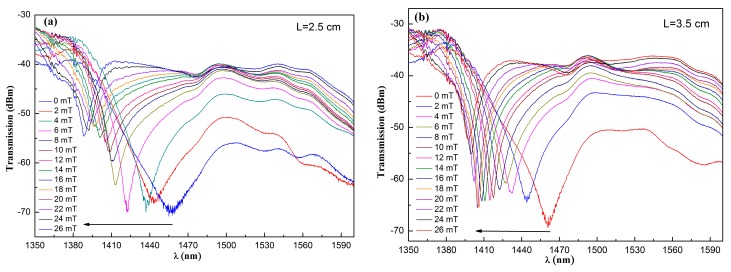

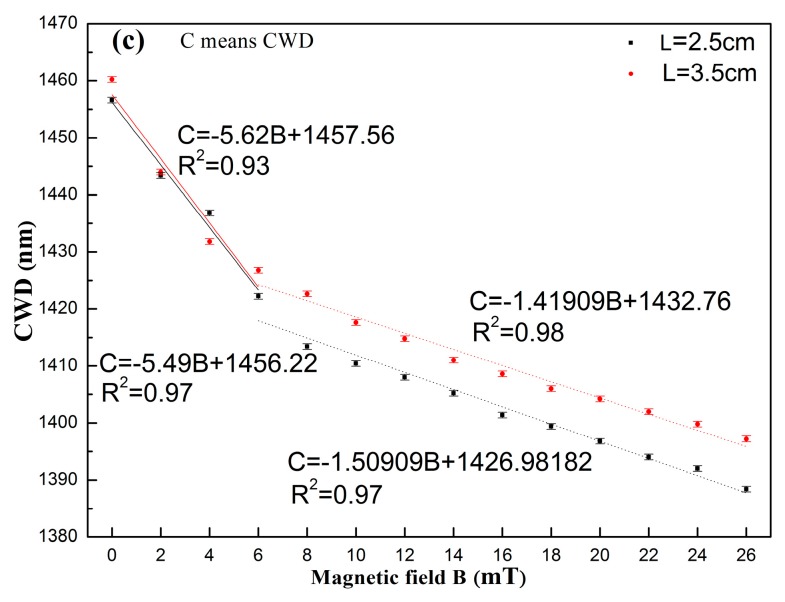
Transmission spectra of the sensing structures consisting of the NCF with lengths of (**a**) 2.5 cm and (**b**) 3.5 cm; (**c**) the relationship between the coupling wavelength dip (CWD) and the magnetic field intensity.

In order to investigate the magnetic field sensing characteristics of the proposed structure in detail, the MF with an RI of 1.45786 is first employed, and two structures consisting of NCF with lengths of 2.5 and 3.5 cm are fabricated. The experimental results are shown in Figure 5. Figure 5a,b display that the CWDs (denoted as C) move to the short wavelength side with the magnetic field (denoted as B), which is contributed to the increase of the RI of the MF with the magnetic field [28]. To be more explicit, the shift of the CWDs labeled in Figure 5a,b with the magnetic field is replotted in Figure 5c. Figure 5c indicates that the CWDs change significantly with the magnetic field under the low field region (≤6 mT). The corresponding sensitivities are 5.49 and 5.62 nm/mT, respectively. As the magnetic field continues to increase, the shift becomes slight. This is due to the saturation magnetization of the MF at a relatively high magnetic field. The corresponding sensitivities at higher field regions (6–26 mT) are 1.51 and 1.42 nm/mT, respectively. The experimental results indicate that the length of the NCF has a negligible effect on the sensitivity of the SNS structure.

**Figure 6:**
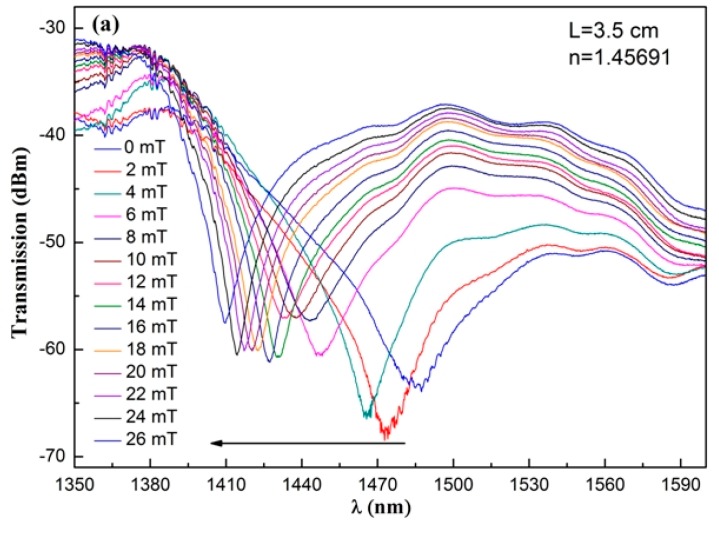

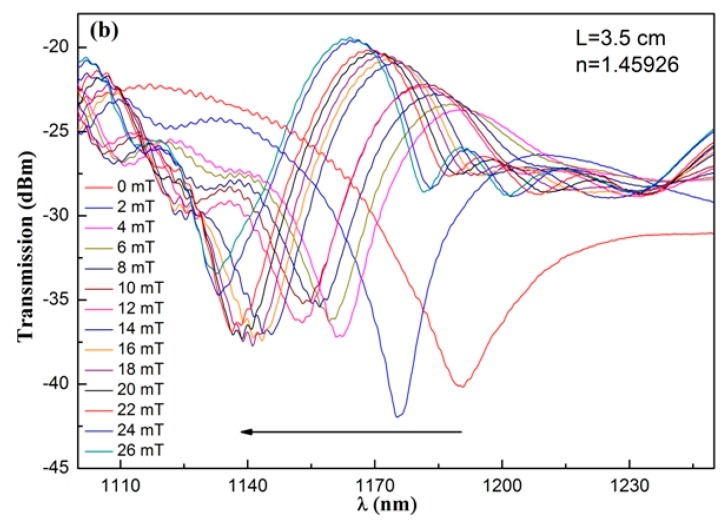
Transmission spectra of the sensing structures with the RI of the MF of (**a**) 1.45691 and (**b**) 1.45926. The length of the NCF is 3.5 cm.

Furthermore, another two SNS structures are fabricated employing MFs with an RI of 1.45691 and 1.45926, respectively. The length of the NCF is kept at 3.5 cm. The very similar experimental results are obtained and shown in Figure 6.

**Figure 7:**
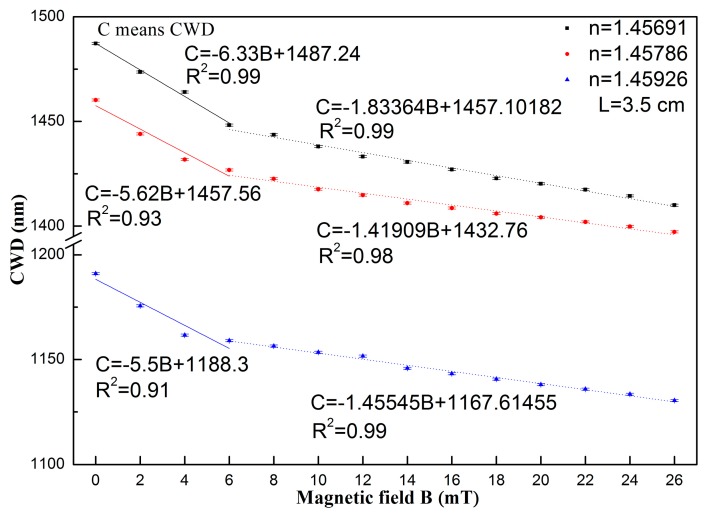
Relationship between the CWD and the magnetic field intensity for the sensing structures covered with an MF of different RIs. The length of the NCF is 3.5 cm.

According to Figure 5 and Figure 6, the relationship between the CWDs and the magnetic field for the three structures with the same length of NCF (3.5 cm) covered with different RIs of the MF (1.45691, 1.45786, and 1.45926) is plotted in Figure 7. From Figure 7, the sensitivities of the three structures are 6.33, 5.62, and 5.50 nm/mT under the low magnetic field region (≤6 mT), respectively. Under the high magnetic field (6–26 mT), the decreased sensitivities of 1.83, 1.42, and 1.46 nm/mT are obtained, respectively. The structure covered with the lowest RI of the MF (1.45691) has the highest sensitivity. This may be due to the dispersion profile difference between the MFs with different RIs. Then, the intersection point of the dispersion profile between the NCF and MF shifts differently for different structures under the same magnetic field change.

Though the low concentration MF is utilized in this work, the sensitivity has been improved significantly compared with those of previously used similar structures, based on mode interference. The highest sensitivity of 6.33 nm/mT was achieved under the low magnetic field region (≤6 mT), which is around 38 times and 7 times higher than those of the very similar SMS (0.1686 nm/mT) and SNS (0.905 nm/mT) structures based on the multimode interference effect [1,6].

## 4. Conclusions

In conclusion, a novel magnetic field sensor based on MF and RIMC is proposed. The traditional SNS fiber structure is employed, but the sensing principle is totally different from the previously employed multimode interference. The length of the NCF has a negligible effect on the sensitivity of the SNS structure. For the as-fabricated structures, the highest sensitivities of 6.33 and 1.83 nm/mT were achieved under the low magnetic field (≤6 mT) and high magnetic field (6–26 mT) regions, respectively. The maximum sensitivity in this work is considerably enhanced compared with those of the previous designed, similar structures. By choosing an MF with high saturation magnetization and designing the initial RI of the MF (under zero magnetic field) with an appropriate value, the sensitivity and sensing range of the proposed magnetic field sensor can be further improved.
